# Supportive Mental Health Self-Monitoring among Smartphone Users with Psychological Distress: Protocol for a Fully Mobile Randomized Controlled Trial

**DOI:** 10.3389/fpubh.2017.00249

**Published:** 2017-09-21

**Authors:** Till Beiwinkel, Stefan Hey, Olaf Bock, Wulf Rössler

**Affiliations:** ^1^Faculty of Business and Economics, Leuphana University of Lüneburg, Lüneburg, Germany; ^2^Movisens GmbH, Karlsruhe, Germany; ^3^Faculty of Business, Economics and Social Sciences, Universität Hamburg, Hamburg, Germany; ^4^Institute of Psychiatry, Laboratory of Neuroscience (LIM 27), University of Sao Paulo, Sao Paulo, Brazil; ^5^Psychiatric University Hospital, Zürich University, Zürich, Switzerland; ^6^Department of Psychiatry and Psychotherapy, Charité – Universitätsmedizin Berlin, Campus Charité Mitte, Berlin, Germany

**Keywords:** mental health, smartphone, mobile intervention, psychological distress, self-monitoring, ambulatory assessment, randomized controlled trial

## Abstract

Mobile health (mHealth) could be widely used in the population to improve access to psychological treatment. In this paper, we describe the development of a mHealth intervention on the basis of supportive self-monitoring and describe the protocol for a randomized controlled trial to evaluate its effectiveness among smartphone users with psychological distress. Based on power analysis, a representative quota sample of *N* = 186 smartphone users will be recruited, with an over-sampling of persons with moderate to high distress. Over a 4-week period, the intervention will be compared to a self-monitoring without intervention group and a passive control group. Telephone interviews will be conducted at baseline, post-intervention (4 weeks), and 12-week follow-up to assess study outcomes. The primary outcome will be improvement of mental health. Secondary outcomes will include well-being, intentions toward help-seeking and help-seeking behavior, user activation, attitudes toward mental-health services, perceived stigmatization, smartphone app quality, user satisfaction, engagement, and adherence with the intervention. Additionally, data from the user’s daily life as collected during self-monitoring will be used to investigate risk and protective factors of mental health in real-world settings. Therefore, this study will allow us to demonstrate the effectiveness of a smartphone application as a widely accessible and low-cost intervention to improve mental health on a population level. It also allows to identify new assessment approaches in the field of psychiatric epidemiology.

## Introduction

Mental health is broadly defined as “a state of well-being in which an individual realizes his or her own abilities, can cope with the normal stresses of life, can work productively and is able to make a contribution to his or her community” (1). Thus, mental health relates not only to the absence of illness but also to positive functioning in regard to well being and social connectedness, and a sense of control and self-efficacy. Indeed, there are strong links between mental health and work productivity, social inclusion, quality of relationships, and life opportunities in general (2–4). There is also a high comorbidity with other health conditions, where poor mental health acts as a risk factor (5). Over the lifespan, mental-health problems often take an episodic course, where relative symptom-free times alternate with more severe episodes of illness (6). Mental ill-health develops frequently in young adults and tends to become chronic in older age if not adequately treated and managed (7).

Mental ill-health is associated with a high societal burden. The most prevalent mental disorders including major depression, anxiety disorders, insomnia, somatoform disorders, and alcohol and drug dependence are among the leading causes of years lived with disability (8). The estimated cost of all mental disorders taken together amounts to up to €240 billion each year in Europe (9). As a result, these countries have made effective, accessible, and high-quality mental-health care a priority, and have called for increasing action to promote mental health in the population (10, 11).

Despite the existence of effective treatment options, improving the mental health of the population is compromised by a low detection rate and treatment-seeking of persons with mental-health problems. Community mental-health surveys indicate that up to 27% of the adult population is affected by at least one mental disorder each year (12) and up to the age of 50, 3/4 of the population has experienced some kind of mental disorder (13). But even in comprehensive health-care systems with free access to mental-health care, as, e.g., in Germany, only up to one-quarter of all persons with mental disorders receive professional help (14). Manifold barriers to help-seeking exist (15), among others also the stigma of mental disorders and self-stigmatization (16, 17). The high numbers of individuals, unaware of their mental-health problem and who consecutively do not seek treatment, lead to the conclusion that a significant amount of unmet treatment needs exist in the population.

Recently, the WHO suggested the use of mobile health (mHealth) technology to improve access to psychological treatment (18). This is referred to as mHealth. Smartphone technology is now widely available in the population, serving as an attractive, but yet under-used delivery channel for health interventions (19). This technology is suitable of repeated sampling of subjects’ current behaviors and experiences in real time and in natural environments, which is referred to as Ambulatory Assessment (AA), Experience Sampling Method, or Ecological Momentary Assessment (20). It can also be combined with psychological interventions, i.e., Ecological Momentary Interventions (EMI). An EMI is a treatment “provided to people during their everyday lives (i.e., in real time) and settings (i.e., real world)” (21). A particularly promising approach for an EMI is the self-monitoring of mental health, where the user learns to keep track of his or her symptoms and behavior over time. Based on the individuals’ real-time information, tailored feedback can be provided to support and reinforce positive change in mental health, and to counteract negative developments. Mobile self-monitoring could not only support mental health but could also stimulate learning about one’s own mental health, and could lead to improved self-management skills and establishment of healthy attitudes and behaviors.

Self-monitoring offers several advantages. First, conducting repeated measurements of mental-health symptoms reveals insights into the dynamics of psychological processes as they naturally develop and this information can be used improve the prediction of relapses or to track treatment response. Second, by establishing short time intervals between experience and recall, or even assessing the present moment, the problem of recall bias, as in retrospective reports, can be avoided. Third, by capturing the real-world contexts, in which experiences are made or behavior occur, risk and protective factors in the psychosocial environment and their impact on mental health can be more easily identified. Fourth, self-monitoring is executed independently, without clinical supervision, which stimulates an active role of the user and empowers subjects to manage their own health. Fifth, self-monitoring can be combined with automated feedback based on the information provided by the individual, which allows for the development of interventions to support mental health and to integrate these interventions in a daily life routine, when support is mostly needed.

Supportive self-monitoring has been successfully used for a broad range of mental-health problems. It can be a feasible, well-accepted approach among patients with mental-health problems (22), and is associated with improvements in clinical outcomes (23–25). Among young adults in the early stages of depression, self-monitoring increases emotional self-awareness, which is an important first step in a stepped care approach (26). Symptom monitoring as part of a disease management program prolonged symptom-free intervals among patients with recurrent depression (27) and helps to focus on that behavior, which patients can influence themselves (28). Among untreated individuals, a motivating effect to seek health care was found (29). Over 50% of persons with mental disorders in all age groups expressed an interest in daily mental health self-monitoring (22). The available evidence on self-monitoring of mental health is limited as most studies are observational and do not employ control groups. Randomized controlled trial (RCT) are needed to investigate benefits and potential harms.

Whether supportive monitoring can be safely applied in unsupervised settings has not yet been sufficiently documented. Besides technical challenges (e.g., battery problems, availability of internet connection), issues of data security and data privacy are critical. As continuous self-monitoring is likely challenging, especially for person with mental-health symptoms, various factors such as low motivation and energy, the tolerability and desirability from the patients’ perspective need to be examined. It is also unclear which subgroups of patients mostly benefit from this type of intervention. Also, the identification and timely management of crises and risk of harm during self-monitoring must be considered. Several trials have shown negative results, including more sustained symptoms as a result of self-monitoring mental health (30). This indicates that self-monitoring needs in-depth consideration and clarification before it is implemented as a clinical tool in the future.

Before this background, we aim to develop and evaluate to the best of our knowledge the first mHealth intervention for mental health on the basis of supportive self-monitoring in the general population. A field test of mental health self-monitoring in the general population has been conducted previously and serves as the basis of this project (31). Building on a smartphone application for Android devices, the mHealth intervention can be disseminated at low cost, and can achieve broad accessibility in the population. Implementing an indicated prevention approach, we want to focus on adults from the general population with psychological distress, who display lowered productivity in day-to-day activities, but who are not seeking professional help. Examining the intervention across subgroups, subjects with low, moderate and high mental distress will be included, covering all degrees of mental-health problems. As smartphones are increasingly used not only by young people, but across all age groups, the study will include young and middle aged adults aged 18–45. We hypothesize that by using the intervention, participant’s mental health and health-related behaviors will improve.

## Material and Equipment

### Stepwise Procedures

A fully mobile 3-arm RCT will be conducted. The intervention will be compared toward AAs (self-monitoring without support) and a passive control group (no self-monitoring and no support). We hypothesize that using the intervention will improve mental health as compared to the control groups, where we expect no significant changes. The RCT will be registered prospectively as a clinical trial in the German registry for clinical trials (Deutsches Register Klinischer Studien).

#### Target Group

The target group for the mHealth intervention are 18- to 45-year-old Android smartphone users with psychological distress. We differentiate between low, moderate, and high psychological distress, as indicated by the Kessler K6 screening scale. The presence of psychological distress as measured by the K6 indicates elevated psychopathology, functional impairment, and treatment need. This was shown in several population-based studies that used the K6 scale as a screening instrument (32, 33). For high distress, a sensitivity of 0.36, specificity of 0.96, and total classification accuracy of 0.92 has been demonstrated in validation studies against structured diagnostic interviews (33). This indicates that subjects with high distress are likely to fulfill diagnostic criteria for a common mental disorder. Participants with moderate distress likely do not fulfill the diagnostic criteria for a mental disorder, but where shown to experience significant psychopathology, functional impairment, and treatment need as well (32).

The size of the target group in Germany can be roughly estimated with a combination of market data on smartphone sales (34, 35), population census data on age distribution (36), and prevalence rates of psychological distress (32). In 2015, about 63% of the German population owned a smartphone, equaling to about 44 million smartphone owners in Germany (34). The leading smartphone operating system is Android with 79.2% of sold smartphones in 2016 (35). Subtracting persons above 45 or under 18 from the 34 million Android smartphone users, we estimate a total of 12.4 million eligible persons who belong to the target group in Germany (36). From the total target group, 63.5% (7.9 million) is estimated to have low psychological distress, 27.9% (3.4 million) is estimated to have moderate psychological distress, and 8.6% (1.1 million) is estimated to have high psychological distress based on the K6 screening scale (32). These estimates indicate that a broad target group exists in the population that could benefit from the mHealth intervention.

#### Recruitment

We aim for a broad recruitment strategy including online and offline channels to achieve a sample that reflects the target group. Online recruitment on social media is a cost-effective approach, which can recruit large numbers of participants in short time, in particular for e-health studies (37, 38). We will place targeted advertisements on Facebook, the most popular social networking platform with 28 million active users in Germany, and Google, the most popular search engine and, with 660 million daily visitors, one of the most frequented websites. Targeted advertisements (e.g., filtered by age, gender, and smartphone usage) ensures that advertisements will reach the target group. While online recruitment is not necessarily superior to offline recruitment, the combination of both approaches was found to be optimal for achieving a representative sample (39). Therefore, efforts will be undertaken to recruit participants in the community, including placing newspaper advertisements, emails to health professionals and civic organizations, media and press releases at Charité Universitätsmedizin Berlin.

#### Sample Size Calculation

A power analysis was performed to detect a group difference on the primary outcome at post-assessment group. The calculation is based on the assumption that a difference between the supported monitoring group and either AA group or control group within the margin −0.25 < Cohen’s *d* < 0.25 can be neglected. We assume no difference between the two latter groups. Therefore, a between-group difference of *d* = 0.25 should reach statistical significance as an indicator of the general effectiveness of the intervention. The target sample size was computed using Gpower (40) for a repeated-measures analysis of variance for a within-between interaction of time and group (ANOVA). We used a targeted power of 95%, a significance level alpha of 0.05, four assessment waves, a conservative estimate of correlation between measurements (*r* = 65) and variances of the differences between groups (non-sphericity correction epsilon = 0.8). This results in a total *N* = 141. Adding 20% drop-out at T2 and T3, we will include 186 participants (62 per group).

#### Sample Description

**Figure 1:**
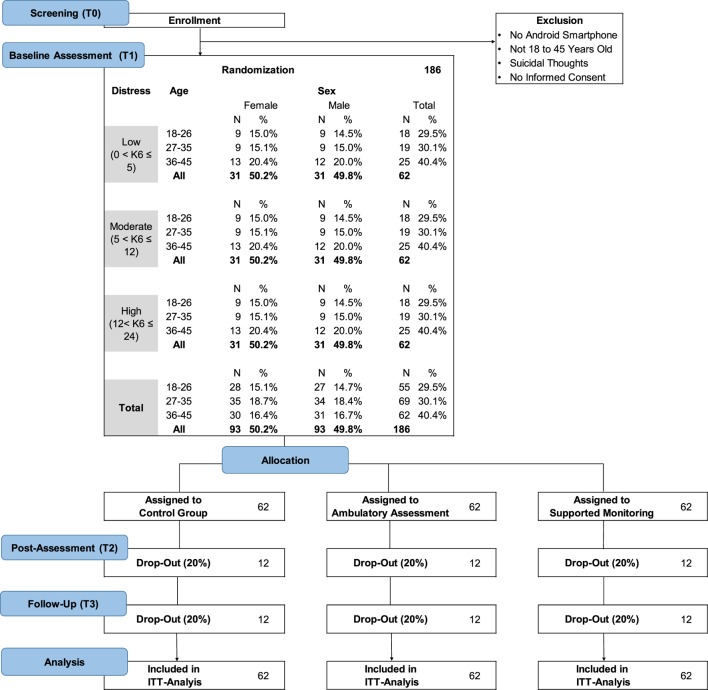
Study flow.

Figure 1 presents the sample description in terms of gender, age, and mental distress. We aim for a quota sample of Android smartphone users from the general population. Quota sampling is a form of non-random sampling which approximates population representativeness according to a set of pre-defined criteria, while allowing for participants to self-select into the study (41). Compared to random sampling or stratified sampling, quota sampling is advantageous for the present project due to lower costs, and because smartphone users need to be pre-screened according to psychological distress.

Three equally sized distress groups are formed, with inclusion probabilities according to prevalence estimates for the Kessler K6 screening scale (32). To achieve equally sized distress groups, participants with moderate to high distress will be over sampled. To approximate representativeness for the general adult population from 18 to 45 years, quotas for age and gender are defined according to the distribution of the latest Zensus population survey (36). Stratification by age is important to account for differential smartphone usage by age, with younger people having a higher rate of smartphone usage as compared to older people. Three age groups will be formed: Young (18–26, 29,5%), Middle (27–35, 30,1%), and Older (36–45, 40,5%). Due to a higher prevalence of mental health problems among women, groups will also be stratified by gender (49.8% male, 50.2% female).

#### Inclusion Criteria, Informed Consent, and Randomization

The inclusion criteria are age between 18 and 45 years and ownership of an Android smartphone (Android Version 4.0 or higher) with an active internet connection. Exclusion criteria are the presence of suicidal thoughts as measured by one item on the presence of suicidal thoughts, and failure to provide informed consent.

Upon registration, participants will be informed about the requirements and procedure of study on the study website. Additionally, an information booklet with details on the study procedure will be available for download. Informed consent will be obtained of each participant with a written signature when first using the smartphone application. To randomize participants to study conditions, a computerized block randomization procedure will be used (Allocation ratio 1:1:1, block size 10). The researcher preparing the randomization procedure will have no information about participants and will not participate in the recruitment, enrollment of participants, or assignment of participants to study groups.

#### Software

Self-monitoring will be conducted on the basis of smartphone application for Android devices. A comparison of such software solutions is provided in Kubiak and Krog (42). MovisensXS will be used as a specialized software for the research methodology of AA, that has been used extensively in research studies (43). MovisensXS is designed to collect subjective self-reports of the participant in daily life and is based on the Android operating system. It consists of two components. First, a web-based portal for study administration used by the researcher. Second, a smartphone app for data collection. The application assesses self-reports throughout the day and, depending on the research question, can be triggered at random times, by the participants themselves or from context parameters.

#### Intervention

The intervention consists of two components: self-monitoring and support.

##### Self-Monitoring

During the 28-day intervention period, daily self-monitoring on the basis of AAs will be conducted. The research methodology of AA is well suited to study people in their natural environment, resulting in ecologically valid data from the persons’ everyday life (44). The self-monitoring protocol should detect and monitor micro-processes of mental health and intra-individual variability, as well as the contextual factors in daily life. We aimed to design a brief, but comprehensive protocol for daily self-monitoring of mental health based on self-reports, while attempting to keep participant burden low. The most common areas of psychopathology will be included: Mood, depression, anxiety, stress, sleep, and functional impairment. In addition, physical activity, social activity, daily hassles and daily uplifts, and alcohol and drug use will be monitored as risk and protective factors of mental health. With a total length of 23 items, participants will be able to complete a daily report in under 5 min. All AAs will be time and date stamped.

*Mood*: To self-monitor daily mood, we will use the version of the Multidimensional Mood State Questionnaire (MDBF) from Wilhelm and Schoebi (45). The MDBF was designed specifically for daily life studies with repeated mood ratings, where a parsimonious measure that is sensitive for change is needed. It uses six items to assess the three factors calmness, valence, and energetic arousal in the present moment. Responses are indicated on three separate six-point bipolar scales with the endpoints labeled “very” (“good–bad,” “awake–tired,” and “calm–nervous”).

*Depression*: depression is one of the most common mental health problems. The Patient Health Questionnaire-2 (PHQ-2) will be used to self-monitor daily depressive symptoms. The PHQ-2 uses two items to score the frequency of depressed mood and anhedonia on the present day on a 4-point scale ranging from (0) “*not at all*” to (3) “*nearly all of today*,” which results in a total score ranging from 0 to 6. The PHQ-2 was originally developed as short screening scale for depression during the past 2 weeks, and will be adapted for the present project to assess depressive symptoms on the present day (46, 47). The PHQ-2 has demonstrated good sensitivity to change and its diagnostic properties were found to be comparable to longer screening instruments. Using PHQ-2 score of 3 as cut point, the authors report that the overall diagnostic accuracy of the PHQ-2 as measured as the “Area under the curve” is 0.90 for major depressive disorder (47); however, the authors also state that a cut point of 2 would enhance sensitivity, and a cut point of 4 would improve specificity.

*Anxiety*: anxiety disorders range over a broad spectrum of experiences related to the expression of anxiety reactions and avoidance behavior. Anxiety is among the most prevalent mental disorders with 11–20% of the population affected during their lifetime. We will use the Generalized Anxiety Disorder-2 (GAD-2) as an ultra-brief monitoring scale for anxiety disorders (48). The GAD-2 uses two items to rate the presence of core anxiety symptoms. The GAD-2 showed high sensitivity for the four most common anxiety disorders: generalized anxiety disorder, panic disorder, social anxiety disorder, and posttraumatic stress disorder. While the original version references to the past two weeks, we will adapt the reference period to the present day. A cut point of 3 on the GAD-2 was reported to be optimal (48).

*Stress*: we define stress as the extent to which an individual perceives that situational demands exceed their ability to cope. To measure the extent of daily stress, we will use the 4-item version of the Perceived Stress Scale (PSS-4). The PSS-4 has two positively worded items and two negatively worded items, which correspond to a two-factor structure (49). We refer to the subscale representing the negatively stated items as “Stress” and to the subscale representing the positively stated items as “Coping.” The PSS uses a 5-point scale from 0 “*never*” to 4 “*very often*,” resulting in a total score of 0–8 for each subscale. As the PSS was not designed as a diagnostic instrument, no cut point is available.

*Daily events*: both daily hassles and daily uplifts will be monitored as risk and protective factors of mental health. Daily hassles are small, day-to-day irritations (e.g., losing things, traffic jam, arguments), while daily uplifts are small, positive events that can help reinforce a sense of well-being (50). The occurrence and appraisal of daily events will be assessed with an adapted scale from Wichers et al. (51), where respondents report the most important event of the day on a 7-point bipolar scale (−3 “very unpleasant,” 0 “neutral,” and 3 “very pleasant”). From this data, variables for positively appraised events (daily uplifts) and negatively appraised events (daily hassles) will be constructed by including the range of neutral to very pleasant events (0–3) or neutral to very unpleasant events (−3–0) events.

*Sleep*: poor sleep quality and insomnia are widespread health problems. To monitor sleeping problems, we will use a one-item measure of sleep quality extracted from the Consensus Sleep Diary, a standardized sleep scale which was designed for self-monitoring purposes (52). The item “How would you rate the quality of your sleep?” is rated on a 5-point sale from 1: very poor–5: very good.

*Physical and social activity*: as a protective factor for mental health, social activity refers to daily interactions with different groups of people in the immediate social environment. It will be measured with the item: “With whom have you spend the majority of your day?” using alone, partner, friends, family, colleagues, and other as categories. Physical activity is monitored as a protective factor of mental health and will be measured with an item extracted from the International Physical Activity Questionnaire (IPAQ SA Short) (53). The item “Have you been physically active today?” uses four answer categories to assess low-, moderate- and high-intensity activities, and also no activity as an indicator for sedentary behavior. Appropriate examples for each category will be provided for instruction (e.g., low-intensity activity “walking at least 10 min a day”).

*Alcohol and drug use*: as a risk factor for psychopathology, daily reports of drinks consumed using the categories beer, wine, liquor, and other will be collected. From this, the number of standard drinks consumed per day will be calculated. Participants will be familiarized with the concept of a standard drink and the volumes of different beverages that are equivalent to a standard drink (54). The German definition of standard drinks will be used (men: 20 g alcohol, women: 10 g alcohol). Two measures of alcohol use will be constructed: days with any drinking and days with heavy drinking (defined by five drinks for men and four drinks in for women). For drug use, the daily occurrence and frequency of any illegal substance will be assessed.

*Impairment*: the individuals’ functioning in the context of social and occupational roles, which is assumed to be a central component of mental health will be monitored. We use a single-item scale from the PHQ-4 (55), which measures subjective impairment in work and household duties and in social relationship on the present day on a 4-point scale with the item: “How difficult have these problems made it for you to do your work, take care of things at home, or get along with other people?”

*Context*: contextual information on daily locations will be monitored as a risk or protective factor of mental health. For this purpose the item “Where were you the majority of the day?” will be used with the categories: at home, visiting friends or family, work or school, outbound, and other.

##### Support

**Figure 2:**
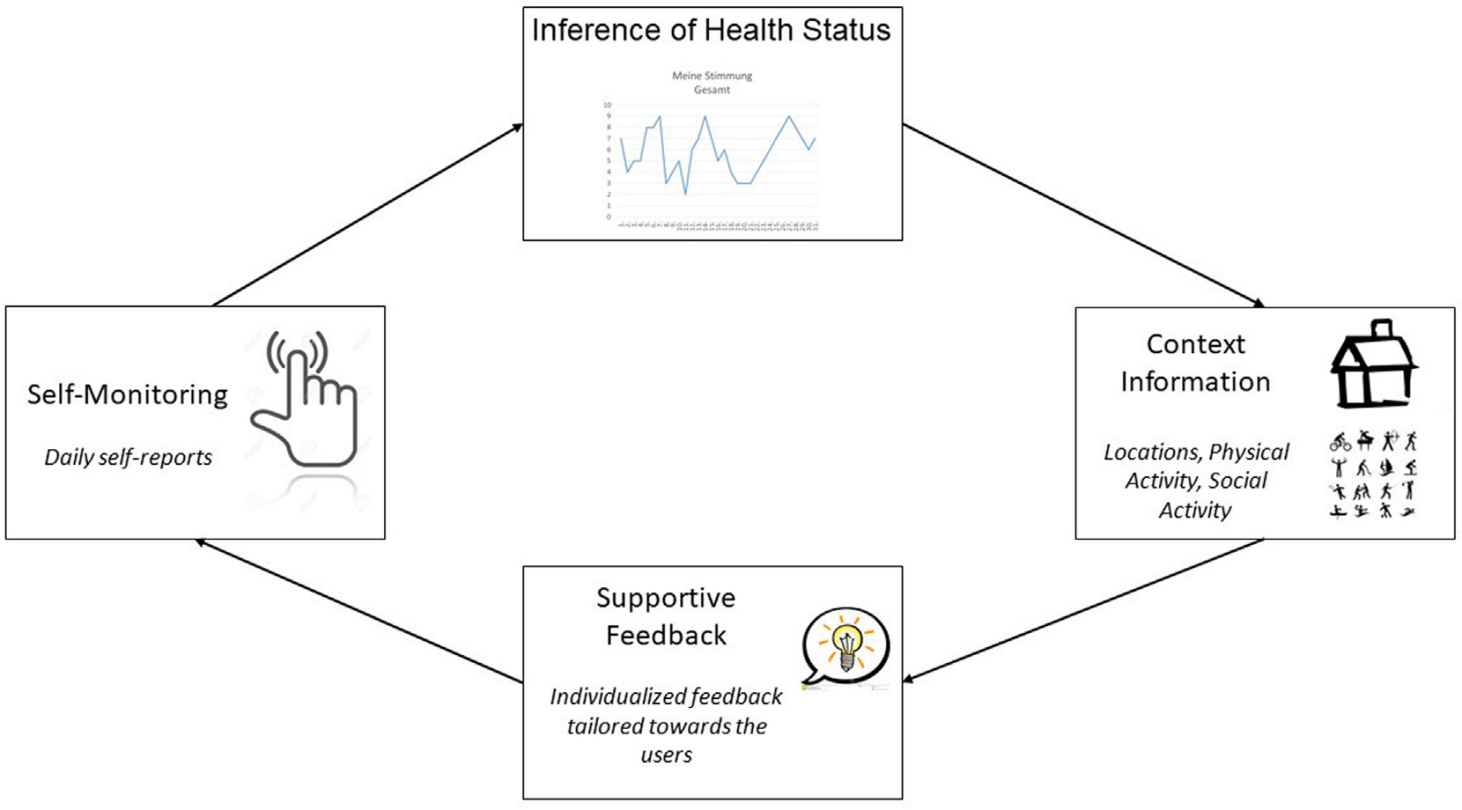
Supported self-monitoring process.

Supportive self-monitoring is a therapeutic intervention to improve mental health, based on tailored real-time feedback on the individuals’ own information collected during self-monitoring (56, 57). By self-monitoring mental health, it is expected that the user gains insights into his or her own mental health, learns to improve self-management skills and establishes healthy attitudes and behaviors. Tailored feedback based on the individuals’ information is provided in real time, to support and reinforce positive change in mental health, and to counteract negative developments. Figure 2 displays the supportive monitoring process.

1. *Self-Monitoring*: participants will be able to self-monitor mental health by visual inspection of the time course for each symptom (mood, anxiety, depression, stress, sleep, impairment). This will be achieved by displaying line plots of symptom scores on the *y*-axis, and time (in days) on the *x*-axis. Participants can select the last week or the total self-monitoring period as time frame. There will be an option to display the time course of multiple symptoms simultaneously by adding or removing symptoms from the menu.
2. *Inference of Health Status*: weekly health reports will provide text-based feedback on the user’s personal health status and symptoms. The participants’ personalized weekly health status will be derived from the mean level for each symptom during the past week. Severity categories (low, moderate, high) will be computed by using established cut points, whenever available. For example, the established PHQ-4 cut points for symptom severity of depression (47) and anxiety (48) will be used. If no cut points are available, the scales will be divided into low, moderate, and high scores according to the tertiles of the frequency distribution. The health status will also be displayed in terms of deviations from the within person-mean. Thus, the participants’ deviation of his or her mean in terms of improvements or worsening of symptoms will be displayed. As a relatively stable mean requires several days of collected data, the latter feature will be available after 1 week of use. Users will be notified whenever a new weekly health report is available with a pop-up message and an icon next to the appropriate section.
3. *Context information*: the influence of social contexts on mental health will be displayed to the user. The context-specific health status will be displayed on a text basis, allowing the users to compare the symptoms and health in each setting. For this purpose, each of seven symptoms will be grouped by its daily context/setting. Six different contexts will be available: 1. days with daily hassles, 2. days with daily uplifts, 3. days with physical activity, 4. days with social activity, 5. days spent alone, and 6. days in company (partner or friends, family, colleagues, others). Alternatively, users may group symptoms by five different locations (1. days at home, 2. days visiting friends or family, 3. days at work or school, 4. trips/outbound, and 5. other). Thus, the user can inspect his or her mental health problems within a total range of 210 possible combination, allowing for a rich and detailed inspection of symptoms by context.
4. *Supportive Feedback*: personalized supportive feedback will be given, based on the data collected by the participant. Feedback will presented in the form of daily health tips which can be accessed from within the application or as Android system notification. The feedback will include tips for the self-management of mental health and mental health micro-interventions (e.g., behavioral activation, problem solving, coping strategies) based on psychoeducation and cognitive–behavioral therapy (CBT) for low mood, anxiety, depression, stress, substance abuse, and sleep problems (58–60). The feedback will be adapted to the severity of each symptom within the last week (low, moderate, high). In the case of low to moderate severity, tips for the self-management of mental health and mental health micro-interventions will be displayed. Participants with moderate symptom severity will also be directed toward available evidence-based self-help programs (61, 62). In the case of severe symptoms, participants will be encouraged to seek help from a professional mental health service and will be provided with corresponding contact information on these services (e.g., hotlines, addresses, and websites of professional organizations). For each mental health problem and degree of severity, at least 10 different feedback messages will be developed, resulting in a total of at least 180 feedback messages (10 messages × 6 mental health problems × 3 degrees of severity). Additionally, a total of 40 feedback messages will be developed for the presence of daily life risk factors for mental ill-health (low social activity, low physical activity, daily hassles, and functional impairment). Users will be able to dismiss feedback messages that they do not wish to be presented again or to select feedback messages that they wish to be reminded of. All content will be developed on the basis of evidence-based guidelines or treatment manuals for the respective mental health problems (63–67).

###### Content

*Psychoeducation*: Psychoeducation aiming at educating the user about the nature and treatment of mental health problems was found to be an effective and easy-to-implement intervention for depression, anxiety, and psychological distress (59). Psychoeducation can also help to reduce perceived stigmatization (68), and to enhance mental health literacy (69), beliefs in the efficacy of treatment options (70), and help-seeking behavior (71). As psychoeducation is most effective when the content is perceived as relevant for a persons’ problem, the intervention will offer relevant information on mental health problems adapted toward the user. This includes psychopathology from self-monitoring (mood, depression, anxiety, stress, sleep, and functional impairment) as well as risk and protective factors. The total educational content will be accessible *via* a knowledge base section within the smartphone application, and supportive feedback (daily health tips) will direct the users toward appropriate educational content, when indicated by self-monitoring information. Psychoeducation within the application will be limited to brief texts and visualizations, and links to further educational websites and resources will be provided for further reference.

*Cognitive–behavioral therapy*: Cognitive–behavioral therapy is one of the most extensively researched forms of psychological treatment. It aims toward improving mental health by challenging negative automatic thoughts and dysfunctional beliefs, and at changing behavioral patterns, including risk and protective factors related to mental health. CBT was found to be effective for a broad variety of mental health problems, including depression, anxiety, and stress (72–74). CBT is also a prevention technique which helps to prevent mental-health problems from maintaining, and is suitable for managing both clinical and subclinical mental health problems (75). CBT is also the recommended therapeutic principle for mHealth interventions, as it is feasible for self-administration by the user (76). In the context of mHealth, CBT has the advantage that its techniques are well suited for a text-based operationalization, allowing CBT to be converted into a short, structured format. Thus, CBT will be the basis for supportive feedback (daily health tips) in the intervention.

*Coping skills training*: as part of CBT-based practices, coping skills training refer to a persons’ perceived ability to effectively cope with adversity and distress, by using adaptive (constructive) coping strategies in stressful situations or during conflicts. Developing active, good coping skills is associated with improved mental health including depression (77), anxiety (78), and psychological distress (79). Improvements in coping skills are also associated with less engagement in avoidance behavior toward potentially anxiety-inducing situations, which in turn leads to increased participation in social activities as a protective factor for the maintenance of mental health (80).

*Behavioral activation*: aiming toward breaking the “vicious cycle” of low mood and inactivity, behavioral activation is a CBT-technique that encourages the user to engage in physically activating and rewarding activities, e.g., by planning of activities, setting goals, and engaging in pleasurable activities. Behavioral activation is associated with improvements in depression (81) and anxiety (82). As part of the daily health tips, we will select short, tangible, and universal activities to maximize user engagement with behavioral activation. By encouraging reflection on the experience after performing an activity, the tips will stimulate reflective learning.

###### Design

To facilitate the adoption and ease of use of the intervention, the smartphone application will be designed toward usability (user-centered design) (83, 84). For example, a tutorial will explain the features and navigation upon initial start of the application. Based on process evaluations, four usability themes for the development of smartphone applications will be considered: design, feedback, navigation, and terminology (85). Design refers to the applications’ interface layout, taking into account consistency, location of icons, and functions on each screen (e.g., the size, color, and esthetics of visual elements). Feedback refers to the applications’ ability to provide appropriate feedback, aimed toward assisting and guiding the user during completion of tasks (e.g., indicating sync status by color when submitting self-monitoring data). Navigation refers to optimizing the way a user navigates throughout the application, so that users know where they are in the application at all times and how to get back to where they came from (e.g., clear icons, tab views, and buttons). Terminology means that the user is able to identify and understand the language used within the application (e.g., avoidance of technical terms).

#### Control Groups

The intervention will be compared to two control groups: first, the active control group will complete daily AAs (self-monitoring without support). Second, there will be a passive control group (no self-monitoring and no intervention).

#### Pilot Test

Prior to recruitment, a pilot test of will be conducted among a sample of 30 students at Charité Universitätsmedizin Berlin, who will receive either course credit or 20€ compensation. All study procedures will be pilot tested.

#### Participant Incentives

To enhance adherence with the study protocol, participants who complete all outcome assessments will receive an incentive of 25€.

Potential “gaming” needs to be avoided, which refers to a situation where research participants fraudulently enroll in a study for the purpose of acquiring research payment. A first measure to prevent gaming is that the eligibility survey will be locked in case of participants trying to change submitted answers. A second measure is that study links will only be available for one user/device. Third, IP addresses will be tracked to prevent duplicate enrollment.

#### Data Storage and Privacy

All data will be stored and handled to fulfill the legal requirements of German data protection. Data is stored in a data center in Greifswald, Germany, which is operated by a German hosting provider (“ProfitBricks GmbH”) certified according to ISO 27001 and Trusted Cloud. To prevent that the identity of the subjects is stored on the server, no person-related data will be collected together with the self-monitoring data (e.g., name). Additionally, a numerical pseudonym label will be assigned, the so-called participant identifier. Participants’ study data can only be linked with person-related data by using the participant identifier. After data collection the participant identifier will be deleted. All communication between smartphone and the server, as well as communication between server and researcher will be encrypted with a 256 Bit Secure Sockets Layer encryption. Finally, to ensure that no unauthorized parties can access the participant data, e.g., in the case of losing the device, all data stored locally on the smartphone will be encrypted with Advanced Encryption Standard 256 bit technology. Each time the participant uses the application, a unique session key is generated to encrypt and securely transmit the data to the server. Only with the private key stored on the server the data can be decrypted. After data transmission from the device to the server, local data stored on the device is deleted.

#### Additional Procedures

*Telephone reminders*: to limit study drop-out, telephone reminders will be initiated in the case of failure to respond to an outcome assessment or failure to download the smartphone application. The study team will constantly supervise enrollment, downloads of application, and compliance with the study protocol, and will initiate telephone calls to participants that need to be reminded.

*Application reminders*: to increase adherence and to stimulate user engagement with the intervention, push notifications on the Android device will remind participants to complete the daily self-monitoring report and will remind users to engage with intervention content, e.g., by displaying a notification that a new daily health tip is available.

### Outcomes

Study outcomes are assessed at four time points: screening (T0), baseline (T1), post-assessment (T3) and follow-up (T4). While the screening (T0) relies on self-assessments collected during participant registration on the study website, structured telephone interviews are conducted at T1, T2, and T3 to assess the primary outcome. The telephone interviews will be conducted by the Academic Survey Lab at the University of Hamburg. Trained interviewers who are blinded to the study condition will conduct the interviews, resulting in an independent outcome assessment to avoid confounding with the self-monitoring data. The secondary outcomes will be self-assessed on the smartphone for all three groups.

#### Primary Outcome

*Mental health*: we will use the extent of psychological distress as a measure of mental health. Psychological distress relates to unspecific behavioral, emotional, cognitive, and psychophysiological components of mental health. The Kessler K6 screening scale will be used (33). The K6 has been extensively used in the World Mental Health Survey and has been validated and translated to German. It consists of six items for the measurement of distress during the past 30 days, resulting in a total score ranging between 0 and 24 points. K6 respondents can be classified into three severity groups, with 0–4 points indicating no distress, 4–12 points moderate distress, and 13–24 points high distress (32).

#### Secondary Outcomes

*Well-being*: the last item from the EUROQOL EQ-5D will be used to measure well-being (86). Participants are asked to rate their well-being on a Likert scale ranging from 0 to 100, where 0 represents the worst state and 100 the best state: “*What number between 0 and 100 best describes your well-being today?*”

*Intentions toward help-seeking*: The intention to seek help for a mental health problem will be measured with the General Help-Seeking Questionnaire (GHSQ) (87). The GHSQ asks: “*If you were having a mental health problem, how likely is it that you would seek help from the following source?”* with two help source options: 1. informal source (friend or family) and 2. professional source (general practitioner, mental health service, educational health service). Respondents rate the likelihood of seeking help from each source on a 7-point Likert scale ranging from “extremely unlikely” to “extremely likely.”

*Help-seeking behavior*: help-seeking is defined as the behavior of actively seeking assistance, regardless of whether the source is informal or formal. The Actual Help-Seeking Questionnaire (AHSQ) will be used to measure help-seeking for a mental health problem within the past 4 weeks (88). The AHSQ first asks whether or not any help had been sought for a mental health problem during the reference period. Second, if help had been sought, whether it was sought from an informal source or from a professional source (identical to the help source options of the GHSQ). For each item, a score of one indicates that help had been sought and a score of 0 that help had not been sought.

*User activation*: we refer to user activation as the degree of perceived knowledge, skill, and confidence for the active self-management of one’s mental health condition. The Patient Activation Measure (PAM) will be used, a reliable and validated measure for the degree of activation. The PAM rates the agreement to 13 items on a 4-point scale (89). Respondents are scored on four levels, with a higher score indicating higher activation.

*Attitudes toward mental health services*: Attitudes about the effectiveness of mental health services influence the likelihood of seeking professional help. The Attitudes Toward Seeking Professional Psychological Help Scale-SF (ATSPPH-SF) will be used to measure attitudes toward mental health services (90). The ATSPPH-SF has 10 items rated on a 4-point scale, resulting in a total score ranging from 0 to 30, where higher values represent more positive attitudes. It has shown good validity and reliability (91).

*Perceived stigmatization*: to measure the extent to which participants perceive to be stigmatized due to a mental health problem, we will adapt the stigma domain of the Caregiver Burden Scale (CBS). The CBS was originally developed to assess four domains of stigma among caregivers of family members with mental illness (92). We only use the domain of stigma which has two items: 1: “*having a mental health problem makes my family feel ashamed”* and 2: “*having a mental health problem makes me feel ashamed*.” Respondents rate the items on a 5-point scale (1 = never, 5 = always). Higher scores indicate a higher level of perceived stigma. A composite measure of the stigma perceived will be constructed from the total of the scores for these two questions.

*Smartphone app quality*: to measure the degree to which the smartphone app meets quality criteria from a user perspective, we will use the Mobile App Rating Scale (MARS) from Stoyanov, Hides (93). The MARS has 23 items and rates smartphone app quality along five dimensions: Engagement, functionality, esthetics, information quality, and subjective quality. It has demonstrated excellent internal consistency and inter-rater reliability. The assessment of smartphone app quality will be conducted at post-intervention among all participants who have used one of the study apps.

*Expectations*: to measure credibility of the smartphone application and the user’s expectations toward it, the credibility and expectation questionnaire (CEQ) will be used. The CEQ consists of six items and has demonstrated good psychometric properties (94).

The following objective usage parameters are automatically collected by smartphone application during the study period.

*Engagement*: the extent to which participants are exposed to the intervention and engage with it will be measured by usage metrics of the smartphone application. This includes a date and time-stamped log of when participants open the application and the length of each session. From this information, the number of and length of user interactions with the application will be computed for the 30-day monitoring period. In the intervention group, the users’ engagement with the supportive feedback content will be assessed by tracking the number of received feedback and user ratings of each feedback message (dismiss/remind).

*Adherence*: adherence with outcome assessments will be measured, and in the groups that use self-monitoring, adherence with self-monitoring will be measured. The number of completed daily self-monitoring will be assessed as defined by completion of the last page of an assessment and can range between 0 and 28.

#### Other Assessments

*Personality*: personality traits will be assessed with the 10 Item Big Five Inventory (BFI-10). The BFI-10 is a short scale to assess the five-factor model as the predominant model for describing personality along the dimensions openness to experience, conscientiousness, extraversion, agreeableness, and neuroticism. The BFI-10 has shown satisfactory psychometric properties (95).

*Optimism/pessimism*: optimisim/pessimism is defined as expectations toward future events in all life domains, with “optimists” having the expectation that mostly good things will happen and “pessimists” having the expectation that mostly bad things will happen. The personality trait optimisim/pessimism will be assessed with the scale Optimism-Pessimism-2 (SOP2). The SOP2 has been validated among the general population and has shown good reliability and validity (96).

*Social support*: the Oslo 3-items social support scale (OSS-3) will be used to measure the extent of available social support. The OSS-3 has been developed as part of a large-scale mental health survey and has been used in European comparative research, where its feasibility and predictive validity with respect to mental health has been confirmed (97).

*Socio-demographics*: core social variables (age, gender, nationality, ethnicity, income, employment status, marriage/relationship status, and education) will be collected on the basis of the recommended measures from the Federal Statistical Office (98).

*Patient history*: past and current intake of medication for mental-health problems, and past use of psychotherapy and similar health services will be assessed. Past use of health services will assess the number and duration of health service usage.

### Statistical Analysis

Before analysis, data will be prepared and cleaned according to the guidance in Ref. (99). Data will then be analyzed according to the recommendations in the CONSORT statement for evaluation of e-health interventions, as well as the mHealth evidence reporting and assessment checklist from the WHO mHealth Technical Evidence Review Group (100, 101). Intention-to-treat analysis (ITT) and additional per-protocol analysis (PP) will be performed, the former including the data of all participants randomized and the latter using only the subset of participants who complete all assessments (T0, T1, T2, T3).

Multilevel models are an appropriate data-analytic strategy for smartphone data, involving daily measurements, resulting in repeated observations nested within individuals (102). These models are well suited to test the general effectiveness of supported monitoring among smartphone users, but also its differential effectiveness and its conditional effectiveness. Models include analysis of both within-person and between-person variance. Missing data will be replaced using Markov Chain Monte Carlo multiple imputation, which is considered to produce more precise estimates of the true intervention in the ITT analysis compared other imputation methods, i.e., last observation carried forward (103).

*General effectiveness*: to test for changes in the outcome variable from before to after the intervention period, repeated-measures analysis of variance (RM-ANOVA) will be conducted for each intervention outcome (104). In the RM-ANOVA, the within-person effect of time is used as an index of general intervention effectiveness. For the analysis of between-person variance, the model can be extended to multivariate analysis of variance (MANOVA). In this case, a between-subjects factor for group membership is added (Supported monitoring group vs. AA group vs. control group). If there is an interaction between the time factor and the group factor, we will conclude that the intervention was effective.

*Differential effectiveness*: to examine person-level moderators of the intervention effect, a between-person factor is added to the MANOVA. This results in a three-way interaction (person factor × time × group). However, as only categorical person variables can be included in the MANOVA, multivariate multilevel models for fixed occasions will also be performed to allow for continuous variables as moderators (105). The between-person moderators to be included in the analysis of differential effectiveness include gender, age, and baseline distress severity (low, moderate, high).

Conditional effectiveness: to examine whether time-varying characteristics of the situation (e.g., days spent at work) or the individual (e.g., being physically active) might moderate the intervention effect, multivariate multilevel models will be extended with a continuous or categorical day-level variable. To estimate the pure within-person effect of the time-varying moderator, continuous day-level variables will be centered on the person-mean.

*Effect sizes*: Cohen’s *d* will be calculated as a measure of the effect size, where *d* = 0.8 or more is classified as a large effect, *d* = 0.5–0.8 is classified as a moderate effect, and *d* = 0.2–0.5 is classified as a small effect. According to Feingold (106), the raw score standard deviation of the outcome variable at baseline assessment will be used to compute Cohen’s *d*.

*Treatment response*: changes in mental health on an individual level will be examined with the widely used Reliable Change Index (RCI) by Jacobson and Truax (107). Participants with an RCI greater than 1.96 will be classified as “responders,” and those with an RCI below −1.96 will be classified as “deteriorated.” To assess the average number of persons who need to be treated to prevent one additional bad outcome, the number needed-to-treat (NNT) will be calculated. Additionally, the number-needed-to-harm (NNH) will be calculated, indicating the number of responders in the intervention group for one extra person to have a symptom deterioration. To set the benefits of the intervention in relation to its risk, the benefit-risk ratio will be calculated by dividing the NNT through the NNH (108).

*Daily life data*: we will compute Cox proportional hazards models to investigate daily life behavior as risk and protective factors of mental health. Cox proportional hazards models are a type of survival model that allows for the estimation of the hazard (or risk) of an event of interest given prognostic variables. We will model changes in mental health as measured by K6 as the outcome event (i.e., improved, maintained, and deteriorated), and separate models will be computed for well-being, intentions to seek help, and help-seeking behavior as outcome event. As risk and protective factors, we will include daily life measures. Risk factors include alcohol and substance use, the frequency of daily hassles, low social activity, and low physical activity. Protective factors include coping behavior, social support, and physical activity. Furthermore, the instability or variability of daily symptoms over the 30-day monitoring period will also be examined as a risk factor by computing within-person variances, first-order autocorrelation, the mean square successive differences, and the probability of acute change according to Jahng et al. (109). Discrimination ability (the ability of the model to separate individuals who develop the event from those who do not) will be assessed with the C statistic. Model calibration, that measure how accurately model predictions match overall observed event rates, will be evaluated with a version of the goodness of fit (110). Internal model validation will be carried out. For all daily life analysis, data from the AA group will be used (*N* = 62).

*Access*: we will describe access to the intervention in terms of population characteristics (sample representativeness), demographics, clinical characteristics, and sample comorbidities using the appropriate descriptive statistics.

*Engagement*: to assess participant engagement, we will examine the proportion of study drop-outs and the proportion of enrolled individuals who respond to the outcome assessments at each time point, and the proportion of daily life assessments filled out during the 30-day interval.

### Trial Status

The trial is in preparation and recruitment will commence after funding has been obtained, approximately in 2018.

## Anticipated Results

This fully mobile RCT will evaluate supportive mental health self-monitoring among smartphone users with psychological distress. Users with moderate to high distress will be oversampled to include all degrees of mental health problems. It compares the intervention toward AAs (self-monitoring without feedback) and a passive control group (no self-monitoring and no intervention). We hypothesize that using the intervention will improve mental health as compared to the control groups, where we expect no significant changes. We will not only test the general effectiveness of the intervention, but also its differential effectiveness (i.e., person-level moderators) and its conditional effectiveness (i.e., time-varying moderators). As the potential risks of using mHealth interventions have not been studied sufficiently, the study will also examine the safety of supportive mental health self-monitoring in regard to detrimental effects on mental health after or during using the application. Finally, the study will provide short-term evidence directly after using the app as well as the long-term sustainability of changes in mental health outcomes. Following current recommendations for evaluating mHealth interventions (100, 101), this study will result in high-quality evidence, allowing for a complete and transparent evaluation of supported self-monitoring on the basis of a smartphone application.

From an epidemiological viewpoint, this study will add important insights into the early development of mental disorders. The existing epidemiological mental health surveys provide good estimates of incidence, prevalence rates, and correlates of mental disorders (111, 112). However, due to their retrospective design, these surveys are limited to a “snapshot” of psychopathology and its correlates. It was found that the time intervals between assessment waves are so long, that there are potentially serious flaws concerning the recall bias (13). Daily life data, as collected during self-monitoring, opens new perspectives for psychiatric epidemiology by capturing the dynamic nature of psychopathology in real-world contexts. Thus, it minimizes the problem of recall bias and maximizes likewise the ecological validity (113). This is especially relevant, considering that mental disorders below clinical thresholds are responsible for a considerable share of the total burden of mental disorders (114), even for more rare mental disorders like psychotic disorders (115). Thus, self-monitoring has the advantage that it is not merely a psychological intervention, but also provides detailed information on the development of mental health over time, related behavior, and the situational context. This information will be used in the proposed study for a comprehensive analysis on risk and protective factors of mental health in daily life.

## Discussion

Despite the substantial burden of mental ill-health on the individual and the economy, there is a significant amount of unmet treatment need in the population. Only about 25% of persons with mental health problems currently receive adequate professional help. To address this treatment gap in mental health care, broadly accessible and evidence-based interventions to monitor and support mental health in daily life are needed. Recently, the use of mHealth technology to improve access to psychological treatment has been suggested. Supportive self-monitoring of mental health in daily life could be a particularly promising mHealth approach. However, additional research is needed to establish supportive self-monitoring as a clinical tool to improve population health in the future.

This study aims to develop and evaluate the first mHealth intervention for mental health on the basis of supportive self-monitoring in the general population. This project will benefit from several lessons learned during previous pilot testing of a smartphone application for mental health self-monitoring (39). The pilot test indicated that mental health self-monitoring is feasible even over longer time periods, if self-monitoring is designed toward minimal participant burden. Pilot testing showed that due to the widespread availability of smartphones in the population, the proposed project does not need proprietary devices to deliver the intervention, rather, the intervention will be available to download on the user’s own Android device. This enables easy access and low-cost distribution. As reaching the target group of smartphone users with psychological distress will be crucial for the present study, a comprehensive, online- and offline-based recruitment campaign will facilitate inclusion. Additional telephone reminders will minimize drop-out during the trial. Blinded outcome assessments by trained telephone interviewers will be conducted, avoiding confounding bias of the intervention outcomes with self-monitoring data, thus providing high-quality data on intervention effectiveness. Additionally, the present study will use objective usage parameters on interaction and engagement with the app to enhance validity. Overall, the proposed project will not only provide new perspectives for psychiatric epidemiology, it also can contribute substantially to the provision of mental health care in the field of early detection and treatment at low costs.

The study has implications for vulnerability-stress models. Vulnerability-stress models seek to explain the onset and development of mental ill-health as a combination of predisposed vulnerability together with acute stress from life experiences (116). Such models highlight the role of environmental stressors, who are assumed to trigger the onset of mental disorders among vulnerable persons. Environmental stressors include the lack of social support or meaningful activities. Vulnerability includes, for example, genetic factors, as well as early life experiences that predispose a person to be more susceptible to a mental disorder. A defining characteristic of vulnerability-stress models is the adoption of a dynamic perspective on the development of mental ill-health, including the investigation of causal factors. Researchers who wish to examine this dynamic perspective empirically cannot achieve this with cross-sectional data, but require longitudinal data. In this context, daily life data collected with mobile-based approaches for self-monitoring of mental health can be used to test different hypothesis derived from vulnerability-stress-models.

Analyzing the time course of daily life data from self-monitoring has the advantage, specific hypothesis on the process of development of mental ill-health can be tested. The “Additity hypothesis” within vulnerability-stress models states a straightforward, linear, dose–response relationship between the accumulated effects of stress at a given time and the sum of vulnerability of a person. Thus, the combined effects of stress and vulnerability are assumed to lead to mental disorder. In contrast, the “Ipsative Hypothesis” states an inverse relationship between the two factors. The greater the presence of one factor, the less of the other is needed to facilitate the onset of mental ill-health. As an outcome for vulnerability-stress models, changes in mental health over the 4-week self-monitoring period could be modeled.

### Limitations

The proposed study has several limitations. First, short screening scales for mental health will be used (self-reports on the device and assessed by telephone interview), but due to feasibility limitations and to minimize participant burden, no diagnostic interview on mental disorders can be conducted (e.g., according to DSM-V).

Second, as participants will self-select themselves into the study, there is potential selection bias. We cannot exclude the possibility that the participants who enroll in the study have certain characteristics that differentiate them from the target group. This means that the generalizability of results is limited to smartphone users who select themselves into using the intervention. However, quota sampling according to age and gender will ensure that the sample will be representative for the population in regard to these characteristics.

Third, potential low response and loss to follow-up present a challenge for this study. Low response/retention rates would reduce the statistical power and can generate a selection bias which affects the generalization of the results. This risk needs to be carefully considered during designing and planning of the study. We will conduct telephone reminders to enhance response. To prevent systematic low response and follow-up loss among hard-to-reach populations, study staff will carefully monitor recruitment rates according to the sample composition in Figure 1: Study flow. Sup-populations showing low response during recruitment will be targeted specifically (e.g., older subjects, male subjects). Using flexible online advertisements, we will place ads that will be shown only to certain sub-populations to enhance response and ensure a representative composition of the sample.

Fourth, potential “gaming” needs to be avoided, which refers to a situation where research participants fraudulently enroll in a study for the purpose of acquiring research payment. Procedures to prevent gaming will include locking the registration procedure in case of participants trying to change submitted answers, availability of study links only for one user/device, and tracking of IP addresses to prevent duplicate enrollment.

Fifth, the event needs to be considered that symptom self-monitoring could have detrimental effects on participants. Appropriate safety measures need to be implemented to counteract participant harm. The study team will consist of a trained staff that can be contacted in the case of a participant experiencing potential detrimental effects. The study team will be available by mail and during telephone hours to provide support, and to forward participants to appropriate mental health-care services.

### Conclusion

mHealth could be widely used in the population to improve access to psychological treatment. This project will not only contribute to the psychiatric epidemiology, but will also allow us to demonstrate the effectiveness of a smartphone application as a widely accessible and low-cost supportive mHealth intervention.

## Ethics Statement

Ethics approval will be obtained from Charité—Universitétsmedizin Berlin.

## Author Contributions

TB drafted the manuscript, WR, SH, and OB contributed to the text and critically revised the manuscript. All authors approved the final version of the manuscript.

## Conflict of Interest Statement

WR, OB, and TB declare that the research was conducted in the absence of any commercial or financial relationships that could be construed as a potential conflict of interest. SH declares to be employed with Movisens GmbH, which will provide the technical platform for the mHealth intervention.

## Supplementary Material

Figure S1 Mock-up of smartphone app.
