# Supplementary figures and images for: Supportive Mental Health Self-Monitoring among Smartphone Users with Psychological Distress: Protocol for a Fully Mobile Randomized Controlled Trial
Source: Front Public Health. 2017 Sep 21;5:249. doi: 10.3389/fpubh.2017.00249 (PMC5613083; doi:10.3389/fpubh.2017.00249)

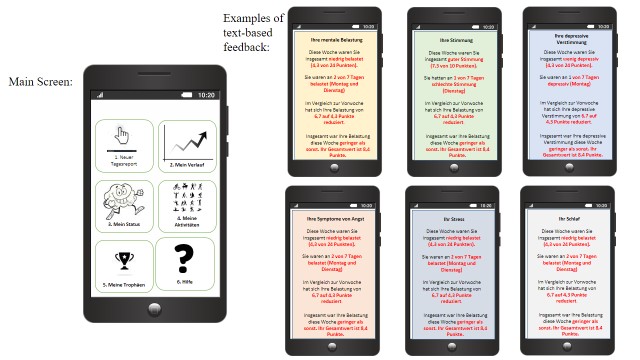

Supplement: Figure S1 — Mock-up of smartphone app. [file Image_1.JPEG]
